# Neuroethics guidance documents: principles, analysis, and implementation strategies

**DOI:** 10.1093/jlb/lsad025

**Published:** 2023-10-26

**Authors:** Matthew R O’Shaughnessy, Walter G Johnson, Lucille Nalbach Tournas, Christopher J Rozell, Karen S Rommelfanger

**Affiliations:** School of Electrical & Computer Engineering, Georgia Institute of Technology, Atlanta, GA, USA; School of Regulation and Global Governance (RegNet), Australian National University, Acton, ACT, Australia; School of Life Sciences, Arizona State University, Tempe, AZ, USA; School of Electrical & Computer Engineering, Georgia Institute of Technology, Atlanta, GA, USA; Emory Center for Ethics Neuroethics Program, Emory University, Atlanta, GA, USA; School of Medicine Departments of Neurology and Psychiatry & Behavioral Sciences, Emory University, Atlanta, GA, USA; Institute of Neuroethics, Atlanta, GA, USA

**Keywords:** neurotechnology, neuroethics, strategy documents, policy, regulation, law

## Abstract

Innovations in neurotechnologies have ignited conversations about ethics around the world, with implications for researchers, policymakers, and the private sector. The human rights impacts of neurotechnologies have drawn the attention of United Nations bodies; nearly 40 states are tasked with implementing the Organization for Economic Co-operation and Development’s principles for responsible innovation in neurotechnology; and the United States is considering placing export controls on brain-computer interfaces. Against this backdrop, we offer the first review and analysis of neuroethics guidance documents recently issued by prominent government, private, and academic groups, focusing on commonalities and divergences in articulated goals; envisioned roles and responsibilities of different stakeholder groups; and the suggested role of the public. Drawing on lessons from the governance of other emerging technologies, we suggest implementation and evaluation strategies to guide practitioners and policymakers in operationalizing these ethical norms in research, business, and policy settings.

## I. INTRODUCTION

Neurotechnologies are poised to drive significant changes to areas from healthcare to human rights. These technologies could transform mobility assistance to those with paralysis, offer interventions for mental health, and drive economic growth. They could also pose new safety and privacy threats, challenge human autonomy, and exacerbate inequality. While emerging technologies that promise broad societal changes are not new, neurotechnologies’ association with the brain creates unique concerns, and scholars and policy bodies have identified significant ethical and policy issues surrounding neurotechnologies.^1^

As such, neurotechnologies have sparked conversations about ethical impacts in a broad range of organizations. For example, the United Nations has recently directed focus toward human rights implications of neurotechnologies,^2^ and the United Nations Educational, Scientific and Cultural Organization is considering the development of a standard-setting instrument on the ethics of neurotechnology.^3^ Nearly 40 national governments have committed to implement the Organization for Economic Co-operation and Development’s nine principles for responsible innovation in neurotechnologies, which calls for both public and private sector action.^4^ Illustrating the prominence of national security concerns, the United States is currently considering placing export controls on brain-computer interfaces.^5^ These nascent discussions and actions have both immediate and long-term implications for researchers, policymakers, the private sector, and society.

In response to these conversations, national and international bodies, academic groups, and technical societies have issued a variety of guidance documents, principles, and frameworks to shape the development and use of neurotechnologies (Table 1). Stakeholders attempting to translate these recommendations into actionable steps need a central access point to this guidance as well as clear strategies for implementation.

**Table 1:** We include English-language documents originally published before February 2022 that provide ethics principles, frameworks, guidelines, and policy strategies for neurotechnology. We include documents written by government or intergovernmental entities as well as documents written by nonprofit, advocacy, and professional organizations, but exclude documents whose authors do not speak as representatives of an organization

**Reference**	**Author/organization**	**Title**	**Date**
*Supra* note 7	American Academy of Neurology Ethics, Law and Humanities Committee	Responding to Requests from Adult Patients for Neuroenhancements: Guidance of the Ethics, Law and Humanities Committee	2009
	*Description:* describes medical, ethical, and legal concerns for physicians prescribing neuroenhancements		
*Supra* note 6	Nuffield Council on Bioethics	Novel Neurotechnologies: Intervening in the Brain	2013
	*Description:* ethical and regulatory frameworks, sensitive applications, and communication strategies		
*Infra* note 9	CeReB: The Center for Responsible Brainwave Technology.	The Ethics of Brain Wave Technology	2014
	*Description:* guidelines for ethical use of neurotechnology by developers		
*Supra* note 7	Consortium of professional clinical groups	Consensus on Guidelines for Stereotactic Neurosurgery for Psychiatric Disorders	2014
	*Description:* discusses ethical structures, consent, and standard of evidence for a clinical audience		
*Supra* note 6, *infra* note 10	US Presidential Commission for the Study of Bioethical Issues	Gray Matters (Volume 1: Integrative Approaches for Neuroscience, Ethics, and Society; Volume 2: Topics at the Intersection of Neuroscience, Ethics, and Society)	2014, 2015
	*Description:* recommendations for US executive branch on key neuroethics topics		
*Supra* note 10	Global Brain Workshop 2016 Attendees	Grand Challenges for Global Brain Sciences	2016
	*Description:* proposes a computational platform and methods to increase cultural awareness		
*Supra* note 6	Morningside Group	Four Ethical Priorities for Neurotechnologies and AI	2017
	*Description:* highlights ethical concerns relevant to neurotechnologies		
*Infra* note 10	Global Neuroethics Summit 2017 Delegates	Neuroethics Questions to Guide Ethical Research in the International Brain Initiative	2018
	*Description:* defines five ‘neuroethics questions’ for researchers conceptualizing and performing research		
*Infra* note 10	US NIH Neuroethics Working Group	Neuroethics Guiding Principles for the NIH BRAIN Initiative	2018
	*Description:* defines eight ‘guiding principles’ for neuroscience research and practice		
*Infra* note 8	The Royal Society Emerging Technologies Working Party	iHuman: Blurring Lines Between Mind and Machine	2019
	*Description:* reviews field; recommends regulatory strategies to advance field and protect public		
*Infra* note 10	US NIH Working Group on BRAIN 2.0 Neuroethics Subgroup	The Brain Initiative and Neuroethics: Enabling and Enhancing Neuroscience Advances for Society	2019
	*Description:* discusses integration of neuroethics into US BRAIN initiative using frameworks of Greely et al. and Rommelfanger et al., *infra* note 10.		
*Supra* note 7	The Japan Neuroscience Society, Ethics and COI Committee	Guidelines for Ethics-related Problems with ‘Non-invasive Research on Human Brain Function’	2019
	*Description:* clinical guidance on noninvasive neuroscience tools based on ethical principles and Japanese law		
*Infra* note 14	Organization for Economic Co-operation and Development (OECD)	Recommendation of the Council on Responsible Innovation in Neurotechnology	2019
	*Description:* establishes nine principles aimed at encouraging ethical use of neurotechnologies by member states		
*Infra* note 8	International Bioethics Committee of UNESCO (IBC)	Report of the IBC of UNESCO on the Ethical Issues of Nanotechnology	2021
	*Description:* findings and recommendations relevant to neurotechnologies’ potential human rights impacts		
*Supra* note 6	Brocher Foundation 2019 Workshop Attendees	Towards a Governance Framework for Brain Data	2022
	*Description:* recommends methods to fill gaps in national and international neurotechnology governance		

In this paper we offer the first review and analysis of existing neuroethics guidance documents (Table 1) recently issued by prominent government, private, and academic groups and offer a deeper discussion of potential implementation strategies. We first examine commonalities and divergences in recent neuroethics guidance documents, focusing on articulated goals, underlying governance frameworks, and the suggested roles and responsibilities of various stakeholders. Drawing on this analysis and lessons from other emerging technologies, we suggest implementation tools and strategies to guide regulatory bodies, policymakers, academia, and the private sector.

## II. ANALYSIS OF DOCUMENTS

### II.A. Articulated Goals, Gaps, and Stakeholders

Guidance documents often justify their development by describing the moral significance and scientific excitement associated with understanding the brain. Brain technologies draw attention due to their current and anticipated potential to enable new medical advances, increase productivity and drive economic growth, and advance national security.

While some documents^6^ offer recommendations for a broad set of stakeholders, others target more specific audiences such as clinicians and physicians,^7^ specific governmental or intergovernmental bodies,^8^ industry,^9^ or the scientific community.^10^ In this sense, documents are presented with a trade-off: those that more narrowly target specific concerns and stakeholder groups often provide the most concrete and actionable recommendations, while those that are broader in scope may lead to less direct action but could be more effective in influencing narratives and agendas.^11^

Each document articulates benefiting the public as a core goal, but documents describe different strategies for achieving this. Some aim to benefit the public indirectly by advancing or protecting the scientific enterprise and field of medicine,^12^ a strategy particularly common in documents targeted toward scientists and clinicians. Others describe principles intended to benefit the public more directly by protecting them from potential harms.^13^

### II.B. Governance Frameworks and Underlying Assumptions

Many documents^14^ derive principles and recommendations from Responsible Research and Innovation (RRI), a science policy framework popularized in the E.U. that emphasizes that science and technology are socially, ethically, and politically interconnected, and that science policy must engage with and be built by a community to serve the community.

Other documents^15^ derive principles and recommendations from field-specific ethical structures. For example, documents targeted to clinicians often adapt medical ethics frameworks such as the Belmont Report and Beauchamps and Childress’ principles of biomedical ethics, while documents targeted to researchers are often derived from bioethics frameworks, placing emphasis on ethics training in education and institutions such as institutional review boards (IRBs).

Two other ideas are commonly used to derive ethical principles. First, documents^16^ commonly reference brain exceptionalism,^17^ the notion that the brain is a uniquely morally salient organ. Second, some documents we analyzed^18^ stress the existence of (and importance of respecting) cultural considerations. These ideas may come into tension with each other: beliefs about agency, autonomy, identity, and the significance of the brain may differ between Western and non-Western cultures.^19^ While documents pointed to the importance of cultural awareness and inclusivity, they offer few directives. This tension echoes debates surrounding human rights, where calls for ‘universal’ values pointing to the pitfalls of ethical relativism come in conflict with a desire to respect diverse cultural norms.^20^

### II.C. Key Topics and Patterns

Key themes, topics, and principles described by documents tend to fall into two major categories. First, documents discuss novel ethical concerns raised by neurotechnologies: safety and privacy; equity and justice; and issues of agency, autonomy, and identity (Table 2). Second, documents discuss procedural and governance concerns that are uniquely or particularly relevant for neurotechnologies (Table 3).

**Table 2:** Key ethics concerns described in guidance documents

**Theme**	**Subtheme**	**Key topics**
Safety and privacy	Safety and risk	Effectively assessing safety and risk
		Sensitive applications (e.g., military/dual use, malign use, manipulation)
		Particular sensitivity due to complexity and significance of the brain
		Particular sensitivity of using neurotechnologies with children
	Privacy	Effective informed consent for data use / privacy concerns
		Use of repurposed data or unexpected future uses
		Reidentification
		Control of data; user ability to amend or delete
		Cultural differences in the importance and meaning of privacy
Equity and justice	Equity and	Equitable distribution of new technologies and therapeutics
	distributive justice	Equitable distribution of risks
		Diversity, inclusion, and avoidance of social/cultural bias in research
		Non-human animal research
	Discrimination and	Protection from brain-data-based discrimination
	stigma	Pressure to use enhancements
		Definitions of ‘normal’ and potential for stigma
Agency, autonomy,	Consent	Meaningful informed consent
and identity		Continuing consent when using technologies that may alter the mind
	Manipulation	Ability of neurotechnologies to manipulate people
		Limiting use in manipulative applications
	Human-ness	Integrity of person
		Regard for donors of, e.g., brain tissue
		Moral significance of synthetically created neural systems

**Table 3:** Key governance concerns described in guidance documents

**Theme**	**Subtheme**	**Key topics**
Public policy	Innovation and regulation	Streamlining/enhancing commercialization pathways
		Drawbacks of shareholder-value motivations
		Licensing issues
		Importance of post-market surveillance
	Limitations of existing governance structures	Regulatory gap between medical and non-medical consumer products
		Difficulty governing concepts involving the brain (e.g., agency)
		Gaps in research ethics, medical ethics, and human rights documents
		Technical capacity of oversight bodies
	Coordination and dialog	Importance of international dialog and cooperation
		Importance of dialog between stakeholders (e.g., between researchers/developers and governance bodies)
		Public participation, education, and dialog
Neuroscience research	Neuroethics in research	Ethics training for scientists
		Building neuroethics infrastructure
		Neuroethics as a tool to both avoid mishaps and engage with societal dimensions of research
		Oversight bodies (e.g., IRBs)
	Accelerating research	Data sharing (e.g., clinical trials, negative results)
		Computational resources and platforms for researchers
Clinical practice	Ethical structures	Patient-physician relationships
		Conflicts of interest
		Distinctions between clinical practice and research
		Oversight bodies (e.g., ethics committees)
		Independent consultations
	Comparisons with	Evidentiary standard to use neurotechnologies
	classical therapies	Potential superiority of ‘low-tech’ methods
		Whether enhancement is a core goal of medicine

#### 1. Ethical Concerns

Highlighted safety concerns include both physical and nonphysical harms related to technologies that interact with—and could modify—the brain in unprecedented ways. Matching these *individual* risks to safety and privacy are *societal* risks concerning equity and justice. Equity concerns focus on cultural biases in research as well as the equitable distribution of both benefits (e.g., novel therapies and enhancements) and harms (e.g., clinical trial subjects). Documents also warn of stigma created when the apparent objectivity of neuroscience research is used to define ‘normality’ and discrimination that could result from the unquestioned use of neuroscience in the courtroom.

Privacy is frequently described as having particular importance for brain data,^21^ with documents citing reidentification risks, unexpected future uses, and concentration of power in data brokers. However, fewer documents^22^ describe major trade-offs inherent to strengthening privacy protections, such as the difficulty of obtaining meaningful informed consent for data collection, cultural variations in notions of privacy, and the costs that restricting data sharing impose on scientific research.

#### 2. Governance and Procedural Concerns

Governance issues highlighted include innovation and regulation policy (such as the need to smooth regulatory pathways for novel therapies), gaps in existing governance structures (for example, gaps in how consumer and medical devices are regulated), and the importance of inter-stakeholder dialog and coordination. Most documents focus on market-based approaches to innovation and governance; a minority of documents^23^ explicitly discuss limitations and constraints of private stakeholders’ responsibility to create shareholder value.

To avoid harmful applications of neurotechnologies, governance-body-oriented documents often focus on horizon scanning capabilities,^24^ while developer-oriented documents often focus on precautionary principles and the need to avoid ‘sensitive’ or ‘malign’ applications (e.g., manipulative marketing, coercion, or military/dual use).^25^

Recommendations frequently cover procedural issues related to neuroscience research and clinical practice. Several documents^26^ articulate a need for neuroethics to be embedded in research and medicine. Documents with other orientations tend to focus more on field-specific oversight mechanisms, such as institutional review boards (IRBs) in clinical trials. Citing the net-societal-benefits of research progress, documents often recommend strategies to accelerate neuroscience research (for example, by enhancing access to shared data and computational resources). Documents also allude to specific ethical issues arising in research and medicine, such as regard for brain donors and non-human animals in research. Finally, in acknowledgement of concerns about hype and hyperbole, some documents with clinical orientations^27^ caution that clinicians should apply a high standard of evidence to novel neurotherapies.

### II.D. Public Participation and Dialog

Guidance documents consistently call for increased communication and dialog with the public. This call is typically framed either as filling a void that might otherwise stimulate misinformation or hyperbole,^28^ or as a more positively-framed attempt to promote civic engagement and dialog.^29^ Many documents^30^ eschew one-way public education efforts in favor of two-way public dialogue, echoing scholarly criticism of the information deficit model of science communication.^31^

Documents ascribe a variety of potential benefits to this two-way public dialogue. One is cultivating public trust in science, an objective alternately framed as respecting public opinion and as avoiding ‘triggering’ public concerns in a way that might reduce public support for neurotechnologies. A second commonly-described benefit of public dialog is building the capacity of the public to engage in debate, shape neurotechnologies’ development, and construct better incentive structures for technology developers. This latter theme is more consistent with RRI frameworks, which call for engagement as a route to meaningful incorporation of public sentiment into policymaking rather than as an outreach exercise alone.^32^ Of particular emphasis is the importance of building capacity to provide informed consent for medical procedures, and the value of neuroscience education for lawyers and judges.

### II.E. Suggested Stakeholder Roles and Responsibilities

One fundamental difference between documents is in their relative emphases on individual responsibility and the need for new incentive and regulatory structures. Documents at one end of this spectrum^33^ portray groups such as neuroscientists and industry as having a powerful self-interest to act responsibly and preserve public trust. Documents closer to the other end of this spectrum^34^ do not place sole ethical responsibility on technology developers, instead calling for governments and other stakeholders to develop incentive structures to encourage prosocial behavior.

#### 1. Clinicians

Physicians are frequently described as having the responsibility to follow their field’s traditional principles for responsible practice and medical ethics. Clinically-oriented documents often^35^ provide physicians with a wide degree of independence and judgment (though others^36^ note the importance in developing clinical consensus).

#### 2. Researchers

Researchers are frequently called on to proactively consider and communicate potential implications of scientific advances. The scientific enterprise in particular is often called upon to improve and meaningfully incorporate ethics in training and the conduct of research, while professional groups are sometimes called on to develop technical standards that can incentivize prosocial outcomes.

#### 3. Government

Governments are often described as representatives of the public,^37^ charged with advancing the interests of underrepresented populations and the natural environment. This results in governments often being tasked with broad goals intended to mitigate the constraints and limitations of other stakeholders. For example, governments are frequently assigned responsibility for addressing equity and justice concerns; wrestling with market forces to steer scientific progress toward societal needs; creating structures for inter-stakeholder dialog and public participation; and implementing horizon-scanning efforts. These vague responsibilities described by broadly-targeted documents contrast with the more concrete but narrowly-scoped recommendations of documents that aim to provide recommendations to specific bodies of governments, which tend to emphasize building technical capacity in government to anticipate ethical concerns; government-led ethical reviews; emphasis on ethics in research funding; and building inter-government and inter-stakeholder dialogs.

#### 4. Funding Bodies

Government funding organizations in particular are often called on to take an anticipatory approach to funding research. Funding agencies are often described^38^ as capable of building incentive structures that steer research trajectories toward societal needs, and are commonly called on to fund ethics training for scientists and integration of ethicists into technical research teams. Research in neuroethics (e.g., on the meaningfulness of consent when therapies may produce personality change) is also cited as an important funding priority.

Many documents^39^ note that consultations and collaborations with ethicists could reduce the need for scientists and developers to have the experience required to anticipate potential ethical implications of their work, a setup that funding agencies are often called on to support. It is also important that scientists working in the private sector have access to consultations and collaboration with ethicists; many companies may invest in legal/compliance groups but lack ethics resources.

#### 5. Media and Public Communicators

One of the most consistent calls^40^ is for stakeholders (e.g., industry, universities, and the media) to perform responsible public communication that avoids hyperbole. Here, too, the field as a whole is often described as having a vested interest in preserving public trust. When more specific recommendations are made, they typically involve the creation of independent or multi-stakeholder bodies responsible for fact-checking and moderating hype.

## III. IMPLEMENTATION STRATEGIES

While these initial ethical norms and recommendations provide an important start, the histories of previous technologies have shown that ethics principles lack actionability without concrete strategies for relevant stakeholder implementation. To be clear, these guidance documents highlight complex challenges without simple solutions. However, while each set of stakeholders faces its own set of political, economic, and process limitations, they also each have unique implementation opportunities. In this section we describe governance tools (Table 4) that stakeholders can use to implement ethics principles, highlighting both strategies that involve binding law as well as non-binding ‘soft law’ mechanisms that provide more flexible tools for steering the development of emerging technologies.^41^

**Table 4:** Implementation strategies for key stakeholders

**Stakeholder**	**Strategies**
Regulatory	Increase regulatory attention toward organizations that collect brain data
bodies	Use healthcare provision to encourage equitable access
	Increase post-market surveillance to bridge the consumer/medical regulatory divide
	Consider more responsive or novel governance strategies
	Utilize international and scientific advisory bodies for horizon scanning, technology assessment, and international coordination
	Build and update international agreements, but not to the exclusion of national and regional governance
	Strengthen incentives for accurate marketing
Clinicians	Accelerate focus on ethics in formal clinical guidance and standards of care
Industry	Develop ESG indicators for neuroethical principles
	Increase attention to ethics in technical standards, industry codes of conduct, and private investment
	Place ethics-based restrictions on how licensees can apply technology
Research enterprise	Increase funding for the development, improvement, and integration of ethics training
	Build and evaluate new models for incorporating ethics expertise in research teams
	Reconceptualize goals of neuroscience to involve sociotechnical concerns
	Advance conversations on data sharing

### III.A. Implementation Strategies for Clinicians

Liability and licensing concerns provide significant incentives for shaping how technologies are used in the clinic. Clinical guidance developed by professional medical societies or licensing authorities can be influential in setting the legal standards of care that inform malpractice case outcomes or licensing decisions, providing these actors with a powerful lever for promoting more ethical uses of new therapeutics. Here, setting standards of care, as well as guidance on informed consent, will be influential in how physicians describe and apply new neurotechnologies. For example, a recent consensus workshop recommends that neuroradiologists who offer inaccurate or biased expert testimony be subject to sanction by their professional society.^42^

### III.B. Implementation Strategies for Industry

Guidance documents nearly universally call for corporate responsibility and stewardship, though robust public and civil society oversight, engagement, and incentive structures (including liability) will likely improve the effectiveness of private sector strategies. Increasingly-popular Environmental, Social, and Governance (ESG) investing strategies may provide one such mechanism; ESG indicators accounting for neuroethical principles could provide for improved regulatory monitoring and more responsible investment. Increased attention to ethics in technical standard-setting, private investment and research funding, industry codes of conduct, and liability schemes may further incentivize corporate stewardship. ‘Ethical licensing’,^43^ in which patent holders place ethics-based restrictions on how licensees can apply new technologies, provides developers a lever to encourage adherence to neuroethical principles.

### III.C. Implementation Strategies for the Research Enterprise

Ethics guideline documents frequently call for the development, improvement, and integration of ethics training into neuroscience. These goals cannot be accomplished without financial support from funding agencies, incentivization by the research enterprise, or mandates from regulatory bodies, including IRBs. New models for incorporating ethics expertise, such as ethics consultancies or ethical standards,^44^ could be valuable in achieving this goal. Researchers and funding agencies can also further technical development of ethics-related research topics such as consent capacity and moral significance; reconceptualizing the goals of neuroscience to involve sociotechnical concerns could help align scientists and ethicists behind these goals.

Academic and private sector researchers are frequently called on to increase data sharing, which can reduce the need for risky clinical trials and speed scientific discovery. This sharing is far more difficult in industrial settings, where intellectual property interests may dominate greater scientific benefits. Further, the benefits of data sharing also come into tension with data protection concerns, and data sharing is particularly difficult in international collaborations where scientists might seek to avoid a web of national data governance laws that place rules on storing data locally or transferring data across borders.^45^

### III.D. Implementation Strategies for Regulatory Bodies

Government bodies often suffer from incomplete or overlapping authority, conflicts between the economic and social interests of innovation, and difficulty in implementing vague principles. However, public bodies also have many regulatory and funding tools at their disposal. To translate ethical principles into action, governments and regulatory bodies can:

#### 1. Treat Brain Data with Special Import

Many companies derive significant value from collecting and processing users’ personal data^46^; because the commercially valuable inferences made from this data may go significantly beyond what users expect, traditional informed consent procedures may be inadequate.^47^ Brain data merit special consideration by regulators. Increased antitrust enforcement and global scrutiny of organizations that collect large amounts of brain data may protect consumers and prevent undesired accumulation of power. Given the sensitive nature of brain data, its use by state and law enforcement also raises concerns about rule of law norms such as due process, governmental search and seizure, nondiscrimination, and freedom of thought.^48^

#### 2. Use Healthcare Provision to Encourage Equitable Access

Guidance documents often task governments with ensuring that new therapeutics (or enhancements) do not exacerbate inequality. Governments can increase access equity by utilizing bodies that make reimbursement decisions, such as the UK’s National Institute for Health and Care Excellence (NICE) or the U.S.’s Medicare and Medicaid, to consider equity when making coverage decisions. While governments can also direct insurers to cover certain new technologies, inequities in insurance coverage mean that countries with universal healthcare schemes have more powerful mechanisms to promote access equity.

#### 3. Strengthen Post-Market Surveillance to Bridge the Consumer/Medical Regulatory Divide

Gaps in the regulatory frameworks used for neurotechnologies marketed for consumer and medical use can allow new products to avoid appropriate regulation and increase the importance of coordination between regulatory bodies.^49^ Post-market surveillance^50^ may provide regulators with increased flexibility to address the unique challenges of nominally consumer-oriented neurotechnology products, though this should not serve as a replacement for appropriate premarket safety evaluations.

#### 4. Consider More Responsive or Novel Governance Strategies

For example, providing regulatory agencies with broader mandates, promoting interagency cooperation, or utilizing sunset provisions for rules may improve responsiveness to broader social interests. Flexible regulatory strategies such as co-regulatory schemes (in which regulators work closely with regulated parties rather than imposing top-down regulation) have been used in nanotechnology and artificial intelligence to guide innovation toward more responsible outcomes.^51^ However, these more flexible approaches often require additional accountability and transparency mechanisms to ensure that private interests do not consume regulatory policy. Market forces may also reduce economic incentives for industry to develop therapies for diseases that are particularly rare or otherwise unprofitable, deficiencies that governments can address through public funding or specialized regulatory pathways such as the U.S. FDA’s Breakthrough Devices Program.

#### 5. Utilize International and Scientific Advisory Bodies for Horizon Scanning, Technology Assessment, and International Coordination

For example, the U.S. National Academies, the UK Royal Society, and the Chinese Academy of Sciences have co-organized international workshops that convened cross-jurisdictional stakeholders to discuss policy implications of heritable human genome editing,^52^ and the World Health Organization has developed a menu of policy options to inform national and international regulation of this technology.^53^ Attempts to move neuroethics guidance from principles to practice could similarly benefit from these types of international and cross-jurisdictional cooperation, alongside the existing efforts of the OECD, UNESCO, and others.

#### 6. Build and Update International Agreements, But Not to the Exclusion of National and Regional Governance

While emerging technologies rarely prompt novel, binding treaties, calls for new and updated human rights in response to neurotechnology developments echo successful (but nonbinding) international instruments such as UNESCO’s 2005 Universal Declaration on Bioethics and Human Rights.^54^ Although these international human rights efforts have important narrative and agenda-setting value, national and regional implementation may be more responsive to local cultural considerations and could have greater long-term legitimacy. Customary international law may offer other routes for developing new legal norms without novel treaties, though this process can take decades. For example, the OECD’s 2019 instrument^55^ and UNESCO’s 2021 report^56^ may provide norms which future international courts could invoke as custom.

### III.D. Implementation Strategies for Public Engagement

Guidance documents almost universally call for increased public outreach and engagement, and some documents suggest concrete strategies.^57^ However, the historical attempts at public engagement about emerging technologies point to both political and financial challenges.^58^

Many guidance documents also call for increasing the quality of public discourse about neurotechnology by improving accuracy and reducing hyperbole. Consumer protection agencies such as the US Federal Trade Commission could strengthen incentives for accurate communication, and lawmakers could consider requiring disclosure of information pertinent to neuroethical norms, steps that could raise the potential for reputational and market consequences for noncompliance with ethical principles (even if no direct legal consequences would result).^59^ Partnerships between researchers and industry that provide the public with trustworthy information could serve the long-term interests of both the public and the field, but care should be taken to meaningfully represent diverse constituents (such as patient groups) and avoid real or perceived capture by industry actors that could erode public trust and moral authority.

### III.E. Evaluating Impact

One route to impact for guidance documents is to make specific, actionable, and politically acceptable recommendations. These specific recommendations provide a clear means for evaluation: are committees, working groups, reports, or rulemaking processes created when called for? Are public engagement events held—and results used to inform policy—when recommended, or is the desire for public participation left as a vague aspiration? Various calls for public engagement prior to the implementation of heritable human genome editing, for example, have yet to yield meaningful policy inputs.^60^

Even when they do not drive direct action, however, guidance documents can achieve impact indirectly by changing values, narratives, and agendas. Though harder to measure, intergovernmental bodies such as the OECD, UNESCO, and Council of Europe have increasingly made explicit references to rights and principles relevant to emerging neurotechnologies^61^; the Chilean legislature recently amended their constitution to add ‘neuro rights’ provisions in response to advocacy by the newly formed NeuroRights Foundation.^62^

Regardless of the route to impact, early signs of the impact of guidance documents may include citations in academic and grey literature or legal documents such as court briefs, judicial opinions, or official transcripts of legislative hearings. Documents cited in technical standards or insurance underwriting are likely to have a larger and more direct impact in technology development, as standards and insurance often serve as a key form of soft law for emerging technologies.^63^ For example, insurers have become quasi-regulators of technologies such as artificial intelligence and nanotechnology as they choose what risk they are willing to absorb and what technological applications they will underwrite.^64^

## IV. CONCLUSION

While emerging neurotechnologies promise significant medical and economic benefits, they also pose novel ethical concerns, from new safety and privacy risks to novel conceptions of human agency and identity. While articulating these concerns is an important first step, it is critical that ethical principles are matched by action. Stakeholders are each constrained by unique obstacles: no governance strategy provides a silver bullet, and successful governance strategies will likely require adaptation over time.

In the face of these challenges, this article presents a set of governance tools to help diverse stakeholders positively shape the impact of neurotechnologies. By beginning to take steps toward governance now, neuroscientists, developers, clinicians, and governance agencies can help translate the ethical principles articulated by guidance documents into positive societal impact.

## FUNDING

This work was supported by The Kavli Foundation (‘Legacy planning for sustainable global neuroethics in the IBI and Beyond’ to K.S.R., which also supported M.R.O, W.G.J., and L.T.), the United States National Institutes of Health (1UH3NS103550 to C.J.R., and 1R01NS115327 to C.J.R., which also supported M.R.O.), and the United States Department of Defense (National Defense Science and Engineering Graduate Fellowship to M.R.O.).

## CONFLICTS OF INTEREST STATEMENT

K.S.R. offers neuroethics consultation to nonprofits and neurotechnology companies. C.J.R. is listed as an inventor on provisional patents for neurotechnologies related to the general topic of this manuscript. The remaining authors declare no financial conflicts of interest.

## Footnotes

1 Goering, S., Klein, E., Specker Sullivan, L., Wexler, A., Agüera y Arcas, B., Bi, G., Carmena, J.M., Fins, J.J., Friesen, P., Gallant, J., Huggins, J.E., Kellmeyer, P., Marblestone, A., Mitchell, C., Parens, E., Pham, M., Rubel, A., Sadato, N., Teicher, M., Wasserman, D., Whittaker, M., Wolpaw, J., Yuste, R, *Recommendations for Responsible Development and Application of Neurotechnologies*, 14 Neuroethics 365–86 (2021).

2 United Nations, *Our Common Agenda: Report of the Secretary General* (2021).

^3^
United Nations Educational, Scientific and Cultural Organization (UNESCO), *Preliminary Study on the Technical and Legal Aspects Relating to the Desirability of a Standard-Setting Instrument on the Ethics of Neurotechnology*. 216 EX/9. Paris, France (2023).

4 Pfotenhauer, S.M., Frahm, N., Winickoff, D., Benrimoh, D., Illes, J., Marchant, G, *Mobilizing the Private Sector for Responsible Innovation in Neurotechnology*, 39 Nat. Biotechnol. 661–4 (2021).

^5^
Borman, M, *Request for Comments Concerning the Imposition of Export Controls on Certain Brain-Computer Interface (BCI) Emerging Technology*, Federal Register, 86: 59070–3 (2021).

^6^
Nuffield Council on Bioethics, *Novel Neurotechnologies: Intervening in the Brain* (2013); U.S. Presidential Commission for the Study of Bioethical Issues, GrayMatters: Topics at theIntersection ofNeuroscience. Ethics, andSociety, Vol. 2 tech. rep. (2015); Yuste, R., Goering, S., Arcas, B.A.., Bi, G., Carmena, J.M., Carter, A., Fins, J.J., Friesen, P., Gallant, J., Huggins, J.E., Illes, J., Kellmeyer, P., Klein, E., Marblestone, A., Mitchell, C., Parens, E., Pham, M., Rubel, A., Sadato, N., Sullivan, L.S., Teicher, M., Wasserman, D., Wexler, A., Whittaker, M., Wolpaw, J, *Four Ethical Priorities for Neurotechnologies and AI*, Nature 551: 159–63 (2017); Ienca, M., Fins, J.J., Jox, R.J., Jotterand, F., Voeneky, S., Andorno, R., Ball, T., Castelluccia, C., Chavarriaga, R., Chneiweiss, H., Ferretti, A., Friedrich, O., Hurst, S., Merkel, G., Molnár-Gábor, F., Rickli, J.M., Scheibner, J., Vayena, E., Yuste, R., Kellmeyer, P, *Towards a Governance Framework for Brain Data*, 15 Neuroethics 20 (2022).

^7^
Larriviere, D., Williams, M. A., Rizzo, M., Bonnie, R. J. & AAN Ethics, Law and Humanities Committee, *Responding to Requests from Adult Patients for Neuroenhancements: Guidance of the Ethics, Law and Humanities Committee*, 73 Neurology 1406–1412 (2009); Nuttin, B., Wu, H., Mayberg, H., Hariz, M., Gabriels, L., Galert, T., Merkel, R., Kubu, C., Vilela-Filho, O., Matthews, K., Taira, T., Lozano, A.M., Schechtmann, G., Doshi, P., Broggi, G., Regis, J., Alkhani, A., Sun, B., Eljamel, S., Schulder, M., Kaplitt, M., Eskandar, E., Rezai, A., Krauss, J.K., Hilven, P., Schuurman, R., Ruiz, P., Chang, J.W., Cosyns, P., Lipsman, N., Voges, J., Cosgrove, R., Li, Y., Schlaepfer, T, *Consensus on Guidelines for Stereotactic Neurosurgery for Psychiatric Disorders*, 85 J. Neurol. Neurosurg. Psychiatry 1003–8 (2014); The Japanese Neuroscience Society, *Guidelines for Ethics-Related Problems with ‘Non-Invasive Research on Human Brain Function’* tech. rep. (2015).

^8^
The Royal Society, *iHuman: Blurring Lines between Mind and Machine* (2019); *International Bioethics Committee of UNESCO (IBC) on the Ethical Issues of Neurotechnology*, SHS/BIO/IBC-28/2021/3 Rev., Paris, France: UNESCO, p. 56 (2021).

^9^

CeReB: The Center for Responsible Brainwave Technology, *The Ethics of Brain Wave Technology* (2014).

^10^

U.S. Presidential Commission for the Study of Bioethical Issues, Gray Matters: Topics at the Intersection of Neuroscience, Ethics, and Society. Ethics, and Society, Vol. 1 (2014); Global Brain Workshop 2016 Attendees, *Grand Challenges for Global Brain Sciences* tech. rep. (2016); Rommelfanger, K.S., Jeong, S.J., Ema, A., Fukushi, T., Kasai, K., Ramos, K.M., Salles, A., Singh, I., Amadio, J., Bi, G.Q., Boshears, P.F., Carter, A., Devor, A., Doya, K., Garden, H., Illes, J., Johnson, L.S.M., Jorgenson, L., Jun, B.O., Lee, I., Michie, P., Miyakawa, T., Nakazawa, E., Sakura, O., Sarkissian, H., Sullivan, L.S., Uh, S., Winickoff, D., Wolpe, P.R., Wu, K.C.C., Yasamura, A., Zheng, J.C., *Neuroethics Questions to Guide Ethical Research in the International Brain Initiatives*, 100 Neuron 19–36 (2018); Greely, H.T., Grady, C., Ramos, K.M., Chiong, W., Eberwine, J., Farahany, N.A., Johnson, L.S.M., Hyman, B.T., Hyman, S.E., Rommelfanger, K.S., Serrano, E.E., *Neuroethics Guiding Principles for the NIH BRAIN Initiative*, 38 J. Neurosci. 10586–8 (2018); NIH Advisory Committee to the Director (ACD) Working Group on BRAIN 2.0 Neuroethics Subgroup (BNS), *The BRAIN Initiative and Neuroethics: Enabling and Enhancing Neuroscience Advances for Society*, National Institutes of Health (2019).

^11^
M. Ienca et al., *supra* note 6; Schiff, D., Biddle, J., Borenstein, J., Laas, K., What’sNext for AI Ethics, Policy, andGovernance? A GlobalOverview inProceedings of the AAAI/ACM Conference on AI, Ethics, andSociety, New York, NY, USA, Feb: ACM, pp. 153–8 (2020).

^12^
Global Brain Workshop 2016 Attendees, *supra* note 10; The Japanese Neuroscience Society, *supra* note 7.

^13^
Nuffield Council on Bioethics; CeReB: The Center for Responsible Brainwave Technology; B. Nuttin et al.; U.S. Presidential Commission for the Study of Bioethical Issues 2014; U.S. Presidential Commission for the Study of Bioethical Issues, *supra* notes 6–10; Organization for Economic Co-operation and Development (OECD), Recommendation of the Council on Responsible Innovation in Neurotechnology, OECD/LEGAL/0457 (2019).

^14^
Nuffield Council on Bioethics; R. Yuste et al.; International Bioethics Committee of UNESCO (IBC); M. Ienca et al., *supra* notes 6, 8; Organization for Economic Co-operation and Development (OECD), *supra* note 13.

^15^
D. Larriviere et al.; CeReB: The Center for Responsible Brainwave Technology; B. Nuttin et al.; NIH Advisory Committee to the Director (ACD) Working Group on BRAIN 2.0 Neuroethics Subgroup (BNS); The Japanese Neuroscience Society, *supra* notes 7, 9, 10.

^16^
Nuffield Council on Bioethics; K.S. Rommelfanger et al.; H.T. Greely et al.; NIH Advisory Committee to the Director (ACD) Working Group on BRAIN 2.0 Neuroethics Subgroup (BNS); The Japanese Neuroscience Society; International Bioethics Committee of UNESCO (IBC); Organization for Economic Cooperation and Development (OECD), *supra* notes 6–8, 10.

^17^
K.S. Rommelfanger et al., *supra* note 13.

^18^
Global Brain Workshop 2016 Attendees; K.S. Rommelfanger et al.; NIH Advisory Committee to the Director (ACD) Working Group on BRAIN 2.0 Neuroethics Subgroup (BNS); Organization for Economic Cooperation and Development (OECD) 2019; International Bioethics Committee of UNESCO (IBC), *supra* notes 7, 10, 13.

^19^
K.S. Rommelfanger et al., *supra* note 13.

^20^
Brown, C, *Universal Human Rights: A Critique*, 1 Int. J. Hum. Rights 41–65: 41 (2017); Donnelly, UniversalHumanRights inTheory andPractice 106–118. Cornell University Press, p. 65 (2013).

^21^
Nuffield Council on Bioethics; CeReB: The Center for Responsible Brainwave Technology; U.S. Presidential Commission for the Study of Bioethical Issues; U.S. Presidential Commission for the Study of Bioethical Issues; R. Yuste et al.; K.S. Rommelfanger; H.T. Greely et al.; NIH Advisory Committee to the Director (ACD) Working Group on BRAIN 2.0 Neuroethics Subgroup (BNS); The Japanese Neuroscience Society; Organization for Economic Cooperation and Development (OECD); International Bioethics Committee of UNESCO (IBC); M. Ienca et al., *supra* notes 6–10, 13.

^22^
H.T. Greely et al.; NIH Advisory Committee to the Director (ACD) Working Group on BRAIN 2.0 Neuroethics Subgroup (BNS); International Bioethics Committee of UNESCO (IBC); M. Ienca et al., *supra* notes 6, 8, 10.

^23^
Nuffield Council on Bioethics; International Bioethics Committee of UNESCO (IBC), *supra* notes 6, 8.

^24^
Nuffield Council on Bioethics; Organization for Economic Cooperation and Development (OECD), *supra* notes 6, 13.

^25^
Nuffield Council on Bioethics; CeReB: The Center for Responsible Brainwave Technology, *supra* notes 6, 9.

^26^
U.S. Presidential Commission for the Study of Bioethical Issues; H.T. Greely et al.; NIH Advisory Committee to the Director (ACD) Working Group on BRAIN 2.0 Neuroethics Subgroup (BNS), *supra* note 10.

^27^
B. Nuttin et al.; U.S. Presidential Commission for the Study of Bioethical Issues, *supra* notes 6, 7.

^28^
Nuffield Council on Bioethics; CeReB: The Center for Responsible Brainwave Technology; H.T. Greely et al.; NIH Advisory Committee to the Director (ACD) Working Group on BRAIN 2.0 Neuroethics Subgroup (BNS); The Japanese Neuroscience Society, *supra* notes 6–8, 10.

^29^
CeReB: The Center for Responsible Brainwave Technology; U.S. Presidential Commission for the Study of Bioethical Issues; U.S. Presidential Commission for the Study of Bioethical Issues; K.S. Rommelfanger et al.; H.T. Greely et al.; The Royal Society; NIH Advisory Committee to the Director (ACD) Working Group on BRAIN 2.0 Neuroethics Subgroup (BNS); Organization for Economic Co-operation and Development (OECD); International Bioethics Committee of UNESCO (IBC), *supra* notes 6–10, 13.

^30^
K.S. Rommelfanger; H.T. Greely et al.; The Royal Society; NIH Advisory Committee to the Director (ACD) Working Group on BRAIN 2.0 Neuroethics Subgroup (BNS); Organization for Economic Co-operation and Development (OECD); International Bioethics Committee of UNESCO (IBC), *supra* notes 8, 10, 13.

^31^

National Academies of Sciences, Engineering, and Medicine, Communicating Science Effectively: A ResearchAgenda, Washington, DC: The National Academies Press (2017).

^32^
Stilgoe, J., Lock, S.J., Wilsdon, J, Why should we promote public engagement with science? 23 PublicUnderstand. Sci. 4–15 (2014).

^33^
D. Larriviere et al.; CeReB: The Center for Responsible Brainwave Technology, *supra* notes 7, 9.

^34^
Nuffield Council on Bioethics; The Royal Society; Organization for Economic Co-operation and Development (OECD); International Bioethics Committee of UNESCO (IBC), *supra* notes 6, 8, 13.

^35^
D. Larriviere et al.; The Japanese Neuroscience Society, *supra* note 7.

^36^
B. Nuttin et al., *supra* note 7.

^37^
Organization for Economic Cooperation and Development (OECD); M. Ienca et al., *supra* note 13.

^38^
U.S. Presidential Commission for the Study of Bioethical Issues; NIH Advisory Committee to the Director (ACD) Working Group on BRAIN 2.0 Neuroethics Subgroup (BNS), *supra* notes 6, 10.

^39^
U.S. Presidential Commission for the Study of Bioethical Issues; H.T. Greely et al.; NIH Advisory Committee to the Director (ACD) Working Group on BRAIN 2.0 Neuroethics Subgroup (BNS), *supra* note 10.

^40^
Nuffield Council on Bioethics; CeReB: The Center for Responsible Brainwave Technology; U.S. Presidential Commission for the Study of Bioethical Issues; H.T. Greely et al.; NIH Advisory Committee to the Director (ACD) Working Group on BRAIN 2.0 Neuroethics Subgroup (BNS); The Japanese Neuroscience Society; Organization for Economic Cooperation and Development (OECD); International Bioethics Committee of UNESCO (IBC), *supra* notes 6, 7, 9, 10, 13.

41 Marchant, G.E., Tournas, L., Gutierrez, C.I., *Governing Emerging Technologies Through Soft Law: Lessons for Artificial Intelligence*, 61 Jurimetrics (2020).

^42^
Meltzer, C.C., Sze, G., Rommelfanger, K.S., Kinlaw, K., Banja, J.D., Wolpe, P.R., *Guidelines for the Ethical Use of Neuroimages in Medical Testimony: Report of a Multidisciplinary Consensus Conference*, 35 Am. J. Neuroradiol. 632–7 (2014).

^43^
Guerrini, C.J., Curnutte, M.A., Sherkow, J.S., Scott, C.T., *The Rise of the Ethical License*, 35 Nat. Biotechnol. 22–4 (2017).

^44^

*Ethically Aligned Design Version 2: A Vision for Prioritizing Human Well-Being with Autonomous and Intelligent Systems* Piscataway, NJ: Institute of Electrical and Electronics Engineers (IEEE) (2019).

^45^
Eke, D.O., Bernard, A., Bjaalie, J.G., Chavarriaga, R., Hanakawa, T., Hannan, A.J., Hill, S.L., Martone, M.E., McMahon, A., Ruebel, O., Crook, S., Thiels, E., Pestilli, F., *International Data Governance for Neuroscience*, 110 Neuron 600–12 (2022).

^46^

Cohen, J.E., Between Truth and Power: The Legal Constructions of Informational Capitalism, 1st edn. Oxford University Press (2019).

^47^

Tournas, L., Neurotechnology: Informed Consent is Working as Designed and That Should Scare Us All, Preprint (2021).

^48^
Farahany, N.A., *Incriminating Thoughts*, 64 StanfordLawRev. 351–408 (2012).

49 Wexler, A., Reiner, P.B., *Oversight of Direct-to-Consumer Neurotechnologies*, 363 Science 234–5 (2019); Johnson, W.G., *Catching Up with Convergence: Strategies for Bringing Together the Fragmented Regulatory Governance of Brain-Machine Interfaces in the U.S.*, 30 Ann. HealthLawLifeSci. 177–206 (2021).

^50^
Fox, B., *Closing the Information Gap: Informing Better Medical Decisionmaking Through the Use of Post-Market Safety and Comparative Effectiveness Information*, 67 FoodDrugLaw J. 83–101, iii (2012).

^51^

Ford, C., Innovation and the State: Finance, Regulation, and Justice, 1st edn. Cambridge University Press (2017).

^52^
National Academies of Sciences, Engineering, and Medicine, In: SecondInternationalSummit onHumanGenomeEditing: Continuing theGlobalDiscussion: Proceedings of aWorkshop inBrief, Olson S. (ed). Washington, D.C.: National Academies Press (2019).

^53^
World Health Organization, HumanGenomeEditing: A Framework forGovernance. Geneva: World Health Organization (2021); World Health Organization, HumanGenomeEditing: Recommendations. Geneva: World Health Organization (2021).

^54^
United Nations Educational, Scientific and Cultural Organization (UNESCO), Universal Declaration on Bioethics and Human Rights tech. repSHS/EST/BIO/06/1, SHS.2006/WS/14, Vol. 11, pp. 377–85 (2006).

^55^
Organization for Economic Co-operation and Development (OECD), *supra* note 13.

^56^
International Bioethics Committee of UNESCO (IBC), *supra* note 13.

^57^
The Royal Society; NIH Advisory Committee to the Director (ACD) Working Group on BRAIN 2.0 Neuroethics Subgroup (BNS), *supra* notes 8, 10.

^58^
J. Stilgoe et al., *supra* note 32; Stilgoe, J., Owen, R., Macnaghten, P., *Developing a Framework for Responsible Innovation*, 42 Res. Policy 1568–80; Kleinman, D.L., Delborne, J.A., Anderson, A.A., *Engaging Citizens: The High Cost of Citizen Participation in High Technology*, 20 PublicUnderstand. Sci. 221–40 (2011).

^59^

Gunningham, N., Grabosky, P.N., Sinclair, D., Smart Regulation: DesigningEnvironmentalPolicy. Oxford; New York: Oxford University Press (1998).

^60^

Greely, H.T., CRISPR People: The Science and Ethics of Editing Humans. Cambridge, Massachusetts: The MIT Press (2021).

^61^
Organization for Economic Co-operation and Development (OECD); International Bioethics Committee of UNESCO (IBC), *supra* note 13; Ienca, M., Common Human Rights Challenges Raised By Different Applications of Neurotechnologies in the Biomedical Field Report Commissioned by the Committee on Bioethics of the Council of Europe (2021).

^62^
Ley No. 21383, Republic of Chile 2021.

^63^
Merchant et al., *supra* note 41.

^64^
Kica, E., Bowman, D.M., *Regulation by Means of Standardization: Key Legitimacy Issues of Health and Safety Nanotechnology Standards*, 53 Jurimetrics 11–56 (2012); Tournas, L.N., Bowman, D.M, *AI Insurance: Risk Management 2.0*, 40 IEEE Technol. Soc. Mag. 52–56 (2021), AI Insurance: Risk Management 2.0.

